# Real‐World Assessment of Economic and Clinical Outcomes in Thai Patients With Respiratory Syncytial Virus Infection Across Age Groups: A Retrospective Cohort Analysis

**DOI:** 10.1111/irv.70039

**Published:** 2024-11-04

**Authors:** Win Khaing, Chia Jie Tan, Chanthawat Patikorn, Chonnamet Techasaensiri, Oraluck Pattanaprateep, Teerapon Dhippayom, Jackrapong Bruminhent, Nathorn Chaiyakunapruk

**Affiliations:** ^1^ Department of Pharmacotherapy University of Utah College of Pharmacy Salt Lake City Utah USA; ^2^ Department of Social and Administrative Pharmacy, Faculty of Pharmaceutical Sciences Chulalongkorn University Bangkok Thailand; ^3^ Department of Pediatrics, Faculty of Medicine Ramathibodi Hospital Mahidol University Bangkok Thailand; ^4^ Department of Clinical Epidemiology and Biostatistics, Faculty of Medicine, Ramathibodi Hospital Mahidol University Bangkok Thailand; ^5^ The Research Unit of Evidence Synthesis (TRUES), Faculty of Pharmaceutical Sciences Naresuan University Phitsanulok Thailand; ^6^ Department of Medicine, Faculty of Medicine Ramathibodi Hospital Mahidol University Bangkok Thailand; ^7^ IDEAS Center Veterans Affairs Salt Lake City Healthcare System Salt Lake City Utah USA

**Keywords:** clinical outcomes, economic outcomes, hospitalization, real‐world assessment, respiratory syncytial virus, Thailand

## Abstract

**Background:**

Respiratory syncytial virus (RSV) is an important cause of acute lower respiratory infections worldwide, including Thailand. This study aimed to assess clinical and economic burdens of RSV infections across different age groups in Thailand.

**Method:**

A retrospective cohort study was conducted using data from a tertiary care hospital from 2014 to 2021. Patients who tested at least one positive RSV were included and stratified into five age groups (< 2, 2–5, 5–18, 18–65, and > 65 years). Healthcare resource utilization, direct medical costs, and clinical outcomes were analyzed with descriptive statistics. Generalized linear models with gamma distributions and log link were used to model cost outcomes. Costs were reported in 2021 US dollars (USD), with 1 USD = 31.98 Thai Baht.

**Results:**

A total of 2122 RSV‐positive patients were identified, half of which (1097) were hospitalized. The median (interquartile range [IQR]) total hospitalization costs ranged from USD780 (IQR: USD488–USD1185) in those < 2 years to USD2231 (IQR: USD1250–USD4989) in those aged 65+ years. Case fatality rates among hospitalized patients also varied from 2.5% to 28.4% depending on age. Increased age, presence of comorbidities, and need for critical care were associated with higher hospitalization costs.

**Conclusion:**

Among RSV‐positive patients, younger children experienced the greatest burden, but poorer outcomes were observed in older adults. Higher costs were associated with older age, comorbidities and critical care needs. Understanding RSV economic burdens is crucial for assessing the cost‐effectiveness and public health value of vaccination programs that prioritize at‐risk groups to mitigate the public health impact.

## 1 Introduction

Respiratory syncytial virus (RSV) is an important cause of acute lower respiratory infections [1]. By the age of 2 years, almost all children have had at least one RSV infection, and half have had two [2]. Infants born at preterm gestation and those with comorbidities (e.g., congenital heart disease and chronic lung disease) are at high risk of severe infection, such as bronchiolitis and pneumonia, which are the leading cause of hospitalization in infants under 1 year old [3]. While RSV most commonly affects infants and children under 2 years of age, it can also pose serious risks for more vulnerable adult populations such as older adults, whom research has identified as another high‐risk group for severe RSV disease. Researchers estimated that 12% of medically attended acute respiratory infections among those over 50 are attributed to RSV [4], and in older adults, an aging immune system and reduced lung function can lead to more severe and prolonged respiratory inflammation following RSV infection [2], with those having chronic medical conditions being even more susceptible.

Globally, the disease burden of RSV includes approximately 20.8 million RSV cases, leading to 1.8 million hospital admissions, 40,000 deaths, and a total of US$611 million in associated direct costs [5]. The annual incidence rates of RSV infection in both healthy older adults and high‐risk individuals in the United States [6] and Europe [7] are approximately 3%–7% in healthy older adults and 4%–10% in high‐risk adults. Among US adults, an estimated 130,000–177,000 hospitalizations [8] and 11,000–17,000 deaths associated with RSV infections occur annually [9, 10]. In the United States, RSV‐related illness results in over 57,000 hospitalizations, 500,000 emergency department visits, and 1.5 million outpatient clinic visits. The mean cost of RSV‐associated illness is over US$130 million annually among children under 5 years old [11].

Most of the RSV burden of disease and costing studies have focused on infants or young children [12, 13, 14]. An estimation of RSV burden and associated costs across age groups remains inconclusive. Based on a recent systematic literature review [15], studies focusing on healthcare resource utilization and costs from RSV across diverse patient age groups, including those with varying comorbidities and age groups outside of US data, are needed. Recently, the economic and clinical burden of RSV infection in pediatric Thai patients under 2 years of age was published [16], the disease burden and economic burden of RSV infection among Thai population across all age population have not been evaluated systematically. During the past decade, RSV tests have been performed regularly at Ramathibodi Hospital. This study was conducted to assess the economic and clinical outcomes in Thai patients with RSV infection across age groups at Ramathibodi Hospital from 2014 to 2021. Findings from this study examining the estimation of healthcare costs associated with RSV will be important to help researchers and policy makers identify populations at highest risk for severe infection and those who cost the most to treat, which can help set immunization program priorities across age groups.

## 2 Methodology

This retrospective cohort study was conducted at Ramathibodi Hospital, Mahidol University, a prominent tertiary teaching hospital located in Bangkok, Thailand. Ramathibodi Hospital maintains a comprehensive patient database that captures demographic, clinical, and financial information for all patients treated at the facility, including details on diagnoses, treatments, procedures, laboratory tests, and associated charges. The study received ethical approval from the Institutional Review Board at the Faculty of Medicine, Ramathibodi Hospital, Bangkok, Thailand (Approval number: COA. MURA2022/234). Informed consent was waived due to the retrospective design of the study. Reporting adhered to the Strengthening the Reporting of Observational Studies in Epidemiology (STROBE) guideline [17].

### 2.1 Patient Population

We identified individuals who underwent at least one RSV test between January 1, 2014, and December 31, 2021. These tests included both rapid antigen and real‐time polymerase chain reaction (PCR) tests, either ordered individually or as part of a broader pathogen panel. Eligible patients were required to have at least one positive RSV test result. The date of the first positive RSV test served as the index date. Subsequently, we collected up to 1 year of follow‐up data, which consist of patient diagnoses, details of healthcare encounters, laboratory tests, medications, and associated charges.

### 2.2 Baseline Variables

Baseline demographic data, including gender, age, and health insurance status at the index date, were extracted from electronic medical records. We identified relevant baseline comorbidities associated with RSV infection using International Classification of Diseases, 10th edition (ICD‐10) codes (see Table S1). These comorbidities included prematurity, perinatal congenital malformations/defects, perinatal respiratory and cardiovascular disorders, neurological disorders, tuberculosis/HIV infections, malignancies, cardiovascular disorders (coronary artery disease, congestive health failure, hypertension), respiratory disorders, Down syndrome, hematological disorders, hepatic disorders, diabetes mellitus, musculoskeletal disorders, nutritional disorders, renal disorders, organ transplant, and pregnancy. Notably, high‐risk factors for RSV infection [18, 19] varied by age group: prematurity was relevant only for children under 2 years old, while perinatal respiratory and cardiovascular disorders, congenital malformations, and defects were exclusive risk factors for those under 18 years old. Solid organ transplants and diabetes mellitus were excluded in children under 2 years old, and pregnancy was recognized as a risk factor for individuals aged 18 and older (see Table S2). Additionally, patients were assessed for coinfection with other respiratory viruses if positive results were documented within 3 days of the index date for influenza virus, adenovirus, human metapneumovirus, seasonal coronavirus, parainfluenza virus, rhinovirus, or bocavirus.

### 2.3 Outcomes

Our study focused on healthcare resource utilization and direct medical costs among RSV‐positive patients from the healthcare provider's perspective. To assess resource utilization, we examined the proportion of patients who received outpatient or emergency care within 14 days of the index date, which corresponds to the average duration of an RSV infection episode. For inpatient care, we analyzed the proportion of patients hospitalized either on or within 14 days of the index date. Additionally, we evaluated the proportion of patients who received critical care (including high‐dependency and intensive care) during their hospitalization. High‐dependency care, referred to as “intermediate care” in the Thai context, provides step‐down medical attention to patients recently discharged from intensive care units but still requiring close monitoring. We also assessed the length of hospitalization (from the index date or admission date, whichever was later) and the duration of stay in critical care units.

Direct medical costs related to outpatient, emergency, and inpatient care based on hospital charges were assessed. For outpatient and emergency care, charges within 14 days of the index date were considered, while for inpatient care, charges during the hospitalization episode were included. All charges were converted to costs using a cost‐to‐charge ratio of 0.73, as outlined in the Ministry of Public Health of Thailand's unit cost manual for hospitals [20]. Additionally, we adjusted all costs for inflation to year 2021 using the method recommended by Turner et al. [21], which involved inflating the local currency using the consumer price index (CPI) from the World Bank [22] and converting to US dollars (USD) using the Bank of Thailand's average exchange rate [23] from Thai Baht to USD. Costs were reported in 2021 USD (1 USD = 31.98 Thai Baht). Total hospitalization costs were calculated by summing all expenses associated with outpatient, emergency, and inpatient care.

Patient clinical outcomes were also evaluated, such as the percentage of patients who experienced RSV‐related clinical complications, including bronchiolitis and pneumonia identified based on ICD‐10 codes within 14 days after the index date. Mortality rates were also retrieved based on documented death dates in inpatient records for patients who died during hospitalization.

### 2.4 Statistical Analysis

Descriptive statistics were used to describe the baseline characteristics, healthcare resource utilization, direct medical costs, and clinical outcomes. Categorical variables were represented using frequencies and percentages, while continuous variables were summarized using mean with standard deviation, additionally, medians and interquartile ranges (IQRs) for skewness data. Generalized linear models with gamma distributions and log link were used to model cost outcomes, considering the typical skewness and heavy tails of financial data. Initial assessments of total hospitalization costs were conducted using univariable models, followed by multivariable models adjusted for age and the presence of RSV‐related comorbidities at baseline, as well as the necessity of critical care unit stay. Additionally, subgroup analyses were performed among hospitalized patients. We stratified patients by age group and critical care status, then calculated median hospitalization costs and length of stay with IQRs for each subgroup. This descriptive approach allowed us to compare outcomes between patients who did and did not require critical care within each age group, providing insights into how age and severity of illness influenced both length of hospitalization and total hospitalization costs. All statistical analyses were conducted using STATA‐SE Version 18 (StataCorp, College Station, TX) [24].

## 3 Results

A cohort of 10,942 patients underwent at least one RSV test between January 1, 2014, and December 31, 2021. Of these, 2122 patients (19.39%) tested positive for RSV and were included in our analysis. Most cases were in the < 2 years age group (*n* = 1370, 64.56%), followed by the 2–5 years age group (*n* = 445, 20.97%). The proportion of elderly individuals (> 65 years) was relatively low (*n* = 133, 14.47%), with the remaining distribution as follows: 18–65 years age group (*n* = 115, 5.43%) and 5–18 years age group (*n* = 59, 2.78%). More than half (53.5%) of the cohort were male. Most patients paid out of pocket without insurance reimbursement (*n* = 862, 40.6%), while almost one‐third ((*n* = 667, 31.4%) and the remaining (*n* = 534, 25.2%) were covered by the Civil Servant Medical Benefits Scheme and the Universal Coverage Scheme, respectively.

Baseline comorbidities differed across age groups. Children < 2 years of age commonly had perinatal issues (8.8%), hematological (6.1%)/respiratory disorders (5.7%), and prematurity (4.4%). Those aged 2–5 commonly had respiratory (9.0%), congenital (6.5%) and neurological disorders (4.9%). Malignancy (20.3%) and hematological disorders (16.9%) were most prevalent in those aged 5–18. Older adults (18–65) commonly had cardiovascular (47.8%)/diabetes disorders (20.9%) while those over 65 also commonly had malignancies (24.1%), respiratory (38.3%), and renal disorders (34.6%). The prevalence of comorbidities increased with age. Coinfection rates with other respiratory viruses at RSV diagnosis also varied by age from 6.4% to 18.8% overall. Table 1 provides detailed patient characteristics.

**TABLE 1:** Baseline demographic and clinical characteristics of patients with RSV positive.

	Up to 2 years	2–5 years	5–18 years	18–65 years	65+ years	Overall
	*n* = 1370	*n* = 445	*n* = 59	*n* = 115	*n* = 133	*n* = 2122
Age, years, median (IQR)	0.94 (0.51, 1.52)	3.35 (2.86, 3.82)	9.07 (6.15, 11.60)	53.89 (39.45, 59.70)	80.49 (72.68, 85.57)	1.61 (0.75, 3.34)
Sex						
Male	761 (55.5%)	244 (54.8%)	35 (59.3%)	45 (39.1%)	50 (37.6%)	1135 (53.5%)
Health insurance at diagnosis, *n* (%)						
CSMBS	371 (27.1%)	139 (31.2%)	10 (16.9%)	48 (41.7%)	99 (74.4%)	667 (31.4%)
UCS	317 (23.1%)	102 (22.9%)	33 (55.9%)	56 (48.7%)	26 (19.5%)	534 (25.2%)
Private	42 (3.1%)	11 (2.5%)	1 (1.7%)	4 (3.5%)	1 (0.8%)	59 (2.8%)
No insurance	640 (46.7%)	193 (43.4%)	15 (25.4%)	7 (6.1%)	7 (5.3%)	862 (40.6%)
Comorbidities, *n* (%)						
Prematurity	60 (4.4%)	NA	NA	NA	NA	60 (2.8%)
Perinatal congenital malformation/defect	120 (8.8%)	29 (6.5%)	2 (3.4%)	NA	NA	151 (7.1%)
Perinatal respiratory and cardiovascular disorder	78 (5.7%)	4 (0.9%)	0 (0.0%)	NA	NA	82 (3.9%)
Neurological disorders	35 (2.6%)	22 (4.9%)	7 (11.9%)	7 (6.1%)	10 (7.5%)	81 (3.8%)
Tuberculosis/HIV infections	6 (0.4%)	6 (1.3%)	0 (0.0%)	10 (8.7%)	3 (2.3%)	25 (1.2%)
Malignancies	14 (1.0%)	16 (3.6%)	12 (20.3%)	38 (33.0%)	32 (24.1%)	112 (5.3%)
Cardiovascular disorders (hypertension and CHF)	40 (2.9%)	16 (3.6%)	9 (15.3%)	55 (47.8%)	106 (79.7%)	226 (10.7%)
Respiratory disorders	45 (3.3%)	40 (9.0%)	5 (8.5%)	17 (14.8%)	51 (38.3%)	158 (7.4%)
Down syndrome	6 (0.4%)	3 (0.7%)	1 (1.7%)	0 (0.0%)	0 (0.0%)	10 (0.5%)
Hematological disorders	83 (6.1%)	23 (5.2%)	10 (16.9%)	17 (14.8%)	16 (12.0%)	149 (7.0%)
Hepatic disorders	25 (1.8%)	5 (1.1%)	3 (5.1%)	13 (11.3%)	12 (9.0%)	58 (2.7%)
Organ transplant	NA	9 (2.0%)	5 (8.5%)	18 (15.7%)	3 (2.3%)	35 (1.6%)
Diabetes mellitus	NA	1 (0.2%)	3 (5.1%)	24 (20.9%)	41 (30.8%)	69 (3.3%)
Musculoskeletal disorders	5 (0.4%)	1 (0.2%)	0 (0.0%)	0 (0.0%)	0 (0.0%)	6 (0.3%)
Nutritional disorders	11 (0.8%)	3 (0.7%)	2 (3.4%)	3 (2.6%)	4 (3.0%)	23 (1.1%)
Pregnancy	NA	NA	NA	5 (4.3%)	0 (0.0%)	5 (0.2%)
Renal disorders	1 (0.1%)	3 (0.7%)	2 (3.4%)	27 (23.5%)	46 (34.6%)	79 (3.7%)
Any of the above	352 (25.7%)	123 (27.6%)	35 (59.3%)	97 (84.3%)	124 (93.2%)	731 (34.4%)
Coinfection with other respiratory viruses at RSV diagnosis, *n* (%)						
Adenovirus	1 (0.1%)	1 (0.2%)	0 (0.0%)	1 (0.9%)	2 (1.5%)	5 (0.2%)
Bocavirus	8 (0.6%)	2 (0.4%)	1 (2.1%)	1 (0.9%)	0 (0.0%)	12 (0.6%)
Human metapneumovirus	6 (0.4%)	3 (0.7%)	0 (0.0%)	2 (1.7%)	0 (0.0%)	11 (0.5%)
Influenza virus	47 (3.4%)	17 (3.8%)	4 (8.3%)	5 (4.3%)	3 (2.3%)	77 (3.6%)
Parainfluenza virus	0 (0.0%)	3 (0.7%)	2 (4.2%)	0 (0.0%)	1 (0.8%)	6 (0.3%)
Rhinovirus	29 (2.1%)	13 (2.9%)	2 (4.2%)	6 (5.2%)	4 (3.0%)	56 (2.6%)
Seasonal coronavirus	3 (0.2%)	2 (0.4%)	1 (2.1%)	4 (3.5%)	0 (0.0%)	10 (0.5%)
Any of the above	88 (6.4%)	39 (8.8%)	9 (18.8%)	17 (14.8%)	9 (6.8%)	165 (7.8%)
Hospitalization						
Yes, *n* (%)	683 (49.85%)	197 (44.27%)	31 (52.54%)	67 (58.26%)	101 (75.94%)	1079 (50.85%)

Abbreviations: CHF, congestive health failure; CSMBS, Civil Servant Medical Benefits Scheme; IQR, interquartile range; RSV, respiratory syncytial virus; UCS, Universal Coverage Scheme; USD, US dollars.

Table 2 presents the healthcare resource utilization, direct medical costs, and clinical outcomes of all eligible patients who tested positive for RSV. Additionally, 203 (19.5%) nonhospitalized patients and 257 (23.8%) hospitalized patients received emergency care. The mean length of hospital stay for hospitalized patients was 11 ± 17 days, with a median of 6 days. Among the hospitalized patients, 257 (23.43%) received critical care, with an average length of stay of 24 ± 43 days. A total of 757 (35.7%) patients developed pneumonia within 14 days of RSV diagnosis. The case fatality rate was higher among hospitalized patients, with 77 (7.1%) deaths compared to 14 (1.3%) deaths among nonhospitalized patients.

**TABLE 2:** Healthcare resource utilization, direct medical costs, and clinical outcomes of patients with RSV positive.

	Nonhospitalized	Hospitalized	Overall
	*n* = 1043	*n* = 1097	*n* = 2122
Healthcare resource utilization			
Patient who received emergency care, *n* (%)	203 (19.5%)	257 (23.8%)	460 (21.7%)
Hospitalization length (days)			
Mean ± SD	NA	11 ± 17	NA
Median (IQR)	NA	6 (4, 10)	NA
Patient who received critical care, *n* (%)	NA	257 (23.43%)	257 (12.11%)
Length of stay in critical care unit (days)			
Mean ± SD	NA	24 ± 43	NA
Median (IQR)	NA	10 (5, 26)	NA
Direct medical costs, USD			
Outpatient costs			
Mean ± SD	111 ± 532	64 ± 144	87 ± 387
Median (IQR)	63 (35, 103)	39 (1, 83)	53 (17, 94)
Emergency care costs			
Mean ± SD	104 ± 183	91 ± 131	97 ± 156
Median (IQR)	37 (26, 68)	38 (8, 105)	38 (17, 89)
Inpatient costs			
Mean ± SD	NA	2187 ± 6270	NA
Median (IQR)	NA	727 (432, 1356)	NA
Total hospitalization costs (outpatient + inpatient + emergency care costs)			
Mean ± SD	131 ± 541	2273 ± 6264	1220 ± 4608
Median (IQR)	66 (44, 113)	814 (500, 1458)	270 (67, 847)
Clinical outcome, *n* (%)			
Patients with respiratory complications	270 (25.9%)	620 (57.5%)	890 (41.9%)
Bronchiolitis	62 (5.9%)	99 (9.2%)	161 (7.6%)
Pneumonia	216 (20.7%)	541 (50.1%)	757 (35.7%)
Case fatality rate among RSV‐positive patients	14 (1.3%)	77 (7.1%)	91 (4.3%)

*Note:* 1 USD = 31.98 Thai Baht.

Abbreviations: NA, not available; RSV, respiratory syncytial virus; USD, US dollars.

The healthcare resource utilization varied across different age groups (Table 3). Specifically, the proportion of patients who received outpatient care before hospitalization ranged from 41.8% in patients aged over 65 years to 80.5% in those aged up to 2 years. Conversely, a smaller proportion of patients up to 2 years old received emergency care (20.6%), compared to 44.6% in those aged over 65 years old. Median hospitalization length varied across age groups from 6 days for those up to 2 years, 4 days for those aged 2–5 years, 6 days for those aged 5–18 years, 9 days for those aged 18–65 years, to longest at 11 days for patients over 65 years. During hospitalization, 22.5% of patients (*n* = 154) in the < 2 years age group, 16.2% (*n* = 32) in the 2–5 years age group, 48% (*n* = 15) in the 5–18 years age group, 27% (*n* = 18) in the 18–65 years age group, and 37.6% (*n* = 38) in the > 65 years age group needed critical care.

**TABLE 3:** Healthcare resource utilization, direct medical costs, and clinical outcomes of hospitalized patients with RSV positive.

	Up to 2 years	2–5 years	5–18 years	18–65 years	65+ years
	*n* = 683	*n* = 197	*n* = 31	*n* = 67	*n* = 101
Healthcare resource utilization					
Patient who received emergency care, *n* (%)	141 (20.6%)	40 (20.3%)	10 (32.3%)	21 (31.3%)	45 (44.6%)
Hospitalization length (days)					
Mean ± SD	9 ± 13	7 ± 10	11 ± 13	20 ± 35	20 ± 25
Median (IQR)	6 (4, 9)	4 (3, 6)	6 (3, 12)	9 (5, 17)	11 (6, 19)
Patient who received critical care, *n* (%)	154 (22.5%)	32 (16.2%)	15 (48.4%)	18 (26.9%)	38 (37.6%)
Length of stay in critical care unit (days)					
Mean ± SD	23 ± 40	19 ± 29	21 ± 26	43 ± 98	21 ± 27
Median (IQR)	9 (5, 24)	8 (3, 18)	11 (4, 33)	11.5 (6, 31)	12.5 (5, 32)
Direct medical costs, USD					
Outpatient costs					
Mean ± SD	56 ± 56	49 ± 65	76 ± 159	125 ± 330	105 ± 330
Median (IQR)	47 (8, 84)	39 (1, 74)	4 (0, 82)	0 (0, 101)	0 (0, 116)
Emergency care costs					
Mean ± SD	30 ± 30	30 ± 39	92 ± 94	253 ± 163	255 ± 165
Median (IQR)	26 (2, 44)	27 (2, 54)	63 (49, 88)	236 (148, 364)	228 (110, 366)
Inpatient costs					
Mean ± SD	1544 ± 4605	1188 ± 3344	4902 ± 11,436	6766 ± 13,816	4615 ± 8056
Median (IQR)	713 (438, 1111)	526 (316, 861)	985 (394, 1815)	1916 (808, 6141)	2019 (768, 4725)
Total hospitalization costs (outpatient + inpatient + emergency care costs)					
Mean ± SD	1605 ± 4598	1245 ± 3339	5007 ± 11,401	6970 ± 13,763	4833 ± 8007
Median (IQR)	780 (488, 1185)	592 (377, 899)	1004 (483, 1862)	2211 (1062, 6962)	2231 (1250, 4989)
Clinical outcome, *n* (%)					
Patients with respiratory complications	439 (64.3%)	106 (53.8%)	11 (35.5%)	22 (32.8%)	42 (41.6%)
Bronchiolitis	87 (12.7%)	11 (5.6%)	0 (0.0%)	0 (0.0%)	1 (1.0%)
Pneumonia	374 (54.8%)	98 (49.7%)	11 (35.5%)	20 (29.9%)	38 (37.6%)
Death among hospitalized patients	20 (2.9%)	5 (2.5%)	6 (19.4%)	19 (28.4%)	27 (26.7%)

*Note:* 1 USD = 31.98 Thai Baht.

Abbreviations: RSV, respiratory syncytial virus; USD, US dollars.

Among hospitalized patients, median total hospitalization costs (outpatient + inpatient + emergency care costs) generally showed an increasing trend with age, ranging from USD780 (IQR: USD488–USD1185) for those under 2 years to USD2231 (IQR: USD1250–USD4989) for those over 65 years, except for ages 2–5 years which had lower median costs of USD592 (IQR: USD377–USD899) compared to those under 2 years. RSV‐related respiratory complications were diagnosed in 33% to 64.3% of the patient cohort across age groups, with case fatality rates among hospitalized patients of 2.9%, 2.5%, 19%, 28%, and 26.7% in < 2, 2–5, 5–18, 18–65, and > 65 years, respectively.

Table 4 showed age‐stratified total hospitalization costs with adjustments for comorbidities and need for critical care. Among hospitalized patients, those with comorbidities had higher hospitalization costs compared to those without (marginal mean difference: USD417; 95% CI: USD219.22–USD616.75). Additionally, patients requiring critical care incurred higher hospitalization costs compared to those without (marginal mean difference: USD4645; 95% CI: USD3830.20–USD5461.02).

**TABLE 4:** Age‐stratified total hospitalization costs (outpatient + inpatient + emergency care costs) adjusted with comorbidities and need for critical care.

Age	Comorbidities		Critical care	
	Marginal mean (95% CI)	*p* value	Marginal mean (95% CI)	*p* value
All hospitalized patients (*n* = 1079)	417.99 (219.22–616.75)	< 0.001	4645.61 (3830.20–5461.02)	< 0.001
< 2 years age group (*n* = 683)	831.98 (591.96–1072.01)	< 0.001	3229.44 (2460.31–3998.58)	< 0.001
2–5 years age group (*n* = 197)	392.21 (127.23–657.18)	0.004	3519.29 (2376.29–4662.29)	< 0.001
5–18 years age group (*n* = 31)	1449.37 (−836.73–3735.46)	0.214	8873.47 (1424.63–16,322.31)	0.02
18–65 years age group (*n* = 67)	−722.75 (−1814.79–369.30)	0.195	13,915.84 (7152.97–20,678.71)	< 0.001
Over 65 years age group (*n* = 101)	104.49 (−419.60–628.59)	0.696	5094.71 (1988.03–8201.39)	0.001

Abbreviations: CI, confidence interval; IQR, interquartile range; RSV, respiratory syncytial virus; USD, US dollars.

The results of our subgroup analyses, presented in Table 5, revealed that patients requiring critical care during hospitalization had higher total hospitalization costs and longer lengths of stay across all age groups. Total hospitalization costs were ranged from a median of USD1862–USD6500. The highest costs were observed in the 18–65 years age group, with a median of USD6500 (IQR: USD2334–USD22,232), and in the over 65 years age group, with a median of USD4855 (IQR: USD2231–USD7702). The longest lengths of stay were observed in the over 65 years age group, with a median of 16 days (IQR: 9–38 days), and in the < 2 years age group, with a median of 14 days (IQR: 8–22 days).

**TABLE 5:** Subgroup analyses of total hospitalization costs (outpatient + inpatient + emergency care costs) and length of hospital stay by critical care requirements.

	Total hospitalization cost, USD		Length of hospitalization, days	
	Median (IQR)	Mean (SD)	Median (IQR)	Mean (SD)
< 2 years age group (*n* = 683)				
Critical care (*n* = 154)	1875.73 (1075.65, 3830.51)	4567.04 (9056.55)	14 (8, 22)	21.18 (23.84)
Noncritical care (*n* = 529)	674.82 (437.52, 929.55)	743.23 (488.18)	5 (4, 7)	5.78 (3.44)
2–5 years age group (*n* = 197)				
Critical care (*n* = 32)	1937.78 (1063.13, 3609.02)	4404.43 (7531.98)	12 (6, 19)	17.34 (17.50)
Noncritical care (*n* = 165)	554.72 (349.55, 757.05)	631.84 (524.14)	4 (3, 5)	4.91 (5.13)
5–18 years age group (*n* = 31)				
Critical care (*n* = 15)	1862.47 (1003.85, 11,948.85)	9580.88 (15,326.51)	12 (5, 27)	17.8 (15.94)
Noncritical care (*n* = 16)	677.48 (372.08, 993.15)	719.89 (438.19)	4 (2, 8)	5.06 (3.80)
18–65 years age group (*n* = 67)				
Critical care (*n* = 18)	6499.95 (2333.95, 22,232.42)	16,753.89 (23,200.10)	12 (8, 42)	38.22 (57.09)
Noncritical care (*n* = 49)	1701.17 (825.49, 3396.50)	3376.42 (4554.13)	8 (3, 14)	13.18 (19.04)
Over 65 years age group (*n* = 101)				
Critical care (*n* = 38)	4854.70 (2230.95, 7702.24)	7980.38 (11,345.73)	16 (9, 38)	27.05 (29.36)
Noncritical care (*n* = 63)	1782.33 (859.91, 2808.39)	2934.91 (4106.37)	9 (6, 15)	15.90 (20.92)

Abbreviations: CI, confidence interval; IQR, interquartile range; RSV, respiratory syncytial virus; SD, standard deviation; USD, US dollars.

## 4 Discussion

This retrospective cohort study from Thailand examined the period from January 2014 through December 2021 and assessed the economic and clinical outcomes of RSV‐infected patients. The results showed that RSV predominantly affected young children under 2 years of age. Male patients accounted for over half of the cases. Baseline comorbidities seemed different across age groups, shedding light on the complex landscape of comorbidities across different age cohorts. Increased age, the presence of comorbidities at the time of RSV diagnosis, and the necessity of critical care were associated with higher hospitalization costs. RSV complications and mortality outcomes also varied across age groups. These findings have addressed the current knowledge gap by providing an overview of the clinical and economic burden of RSV infection across different age groups in Thailand.

Our findings indicate that comorbidities in RSV‐infected hospitalized patients varied significantly across age cohorts, reflecting how different health conditions among various age groups affect susceptibility to and severity of RSV infection. Comorbidity profiles changed substantially from early childhood to elderly age groups. Specifically, higher comorbidities and coinfection were observed in adults (18–65 years) and seniors (> 65 years) when compared to children (< 18 years). This pattern aligns with other studies from Germany [25] and Korea [26], which similarly reported age‐related increases in underlying chronic conditions including cancers and complications in patients with RSV infection. In addition, study by Falsey et al. [6] demonstrated that RSV in older adults could lead to severe pneumonia and a high rate of hospitalization.

The need for critical care and the higher mortality rates in adult and senior patient groups align with findings from the Centers for Disease Control and Prevention (CDC), which indicate that older adults with RSV are at a heightened risk of severe illness [27]. These observations underscore the importance of targeted prevention strategies, including vaccination and prompt antiviral treatment for older adult and senior populations. Additionally, they highlight the need for increased vigilance in managing adults and seniors with RSV, perhaps integrating proactive monitoring for those with known chronic conditions that may exacerbate the impact of RSV infection in older Thai patients.

Furthermore, we found that increased age, the presence of comorbidities, and the need for critical care services each contributed significantly to a higher economic burden as measured by hospitalization costs. These cost drivers have been consistently recognized in previous studies assessing RSV‐associated healthcare spending. McLaurin et al. [14] discovered that in the United States, the mean costs of RSV hospitalizations were approximately $8324 for full‐term infants, escalating to 4.4 times higher for those admitted to the ICU and further increasing by 1.5–2.5 times for infants requiring mechanical ventilation compared to those in the ICU. The study by Choi et al. [28] in the United States identified predictors of higher costs, which included chronic liver disease, a length of stay of four days or more, and antibiotic use (in ICU admissions) in patients over 18 years old. Higher costs in comorbid or critically ill patients can be attributed to treatment intensification, prolonged hospital length of stay, and the consumption of expensive resources like ventilation support. Patients in critical care who require mechanical ventilation have been shown to incur upwards of a 94% average cost increase due to mechanical ventilation in respiratory system diseases [29]. Our results, therefore, align with existing literature, demonstrating that patients with clinical complexities or severity of illness due to factors such as critical illness and comorbid disease pose a greater economic burden on healthcare systems when hospitalized with RSV infection.

Our study helps provide important insights into the economic burden of RSV that are highly relevant given the recent approval and availability of new RSV vaccination options. Specifically, our analysis adds to the limited data on RSV costs in Thailand and can serve as a model for estimating national economic burden in other Southeast Asian countries where such data are often lacking. Understanding the cost implications of RSV at both the hospital and national level is critical for assessing the potential value and cost‐effectiveness of new RSV vaccines from the perspective of the Thai healthcare system. Integrating our cost estimates with national epidemiological data, if available, could help provide a more comprehensive picture of the overall fiscal impact of RSV disease. Our study highlighting the increasing economic burden with age further supports the CDC's recent guidance to consider RSV vaccination for older adults based on individual risk–benefit assessments. As vaccination options expand globally, more cost analyses contextualized for low‐ and middle‐income country healthcare settings will be important to guide public health recommendations and resource allocation decisions regarding RSV prevention.

Our study has some limitations. Firstly, we identified patients based on positive RSV tests, which are typically ordered for those with severe symptoms or risk factors. Consequently, less severe RSV cases may have been excluded, potentially overestimating resource use and costs. Secondly, our study focused solely on direct medical costs from patient databases. We could not capture nonmedical expenses (like transportation or food) or indirect costs (such as caregivers productivity loss) or RSV‐specific medication costs. Thirdly, the reason for increased hospitalization costs in patients with many comorbidities was uncertain because we could not break down the cost (e.g., antibiotic use or other comorbidity‐related pharmacy costs) during critical care. Fourthly, the small sample sizes for patients aged 5–18 and 18–65 years, limiting the precision of cost estimates in those groups. As data were collected from a single large medical center where RSV testing is commonly performed, results may have limited generalizability to other medical facilities in Thailand where RSV testing is not routinely conducted. Additionally, while we adjusted for inflation, we did not account for potential complexities in hospital protocols during the COVID‐19 pandemic, which could impact costs. Finally, our study highlighted the overall impact of comorbidities adjusted with Charlson Comorbidity Index score on hospitalization costs across all age groups. However, future studies should identify comorbidity patterns associated with the highest hospitalization costs across different age groups and report the hospitalization cost separately for patients with no comorbidities, those with single comorbidities, and those with multiple comorbidities.

## 5 Conclusion

In conclusion, RSV infections in Thailand significantly impacted healthcare resources and costs, especially in those under 2 years of age and those over 65. Younger children had the highest burden while older adults had poorer outcomes. Higher costs were seen in older patients, those with comorbidities, and those requiring critical care. Understanding the economic burden associated with RSV will be important for evaluating whether implementation of RSV vaccines at the population level represents an efficient allocation of healthcare resources from a public health perspective. Identifying at‐risk groups and prioritizing vaccinations can help mitigate the public health impact of severe RSV disease.

## Author Contributions

**Win Khaing:** formal analysis, writing – original draft, methodology, writing – review and editing, visualization. **Chia Jie Tan:** data curation, formal analysis, writing – review and editing, validation. **Chanthawat Patikorn:** data curation, writing – review and editing, validation. **Chonnamet Techasaensiri:** writing – review and editing, supervision, resources, conceptualization. **Oraluck Pattanaprateep:** resources, writing – review and editing. **Teerapon Dhippayom:** writing – review and editing. **Jackrapong Bruminhent:** writing – review and editing. **Nathorn Chaiyakunapruk:** conceptualization, visualization, writing – review and editing, supervision, project administration, methodology.

## Conflicts of Interest

The authors declare no conflicts of interest.

### Peer Review

The peer review history for this article is available at https://www.webofscience.com/api/gateway/wos/peer‐review/10.1111/irv.70039.

## Supporting information


**Table S1** ICD‐10 codes of high‐risk conditions for patients with respiratory syncytial virus infection.
**Table S2** High‐risk categories for patients with respiratory syncytial virus infection by age group.

## Data Availability

The data are not publicly available. Regarding the data, please reach out to the corresponding author, Nathorn Chaiyakunapruk (nathorn.chaiyakunapruk@utah.edu).
