# Supplementary material for: Real‐World Assessment of Economic and Clinical Outcomes in Thai Patients With Respiratory Syncytial Virus Infection Across Age Groups: A Retrospective Cohort Analysis
Source: Influenza Other Respir Viruses. 2024 Nov 4;18(11):e70039. doi: 10.1111/irv.70039 (PMC11534647; doi:10.1111/irv.70039)
Supplement: Supplementary file 1 — Table S1 ICD‐10 codes of high‐risk conditions for patients with respiratory syncytial virus infection. Table S2 High‐risk categories for patients with respiratory syncytial virus infection by age group. [file IRV-18-e70039-s001.docx]

# Supplemental Table 1. ICD-10 codes of high-risk conditions for patients with respiratory syncytial virus infection

| **High risk category** | **Medical conditions** | **ICD** |
| --- | --- | --- |
| Prematurity | Disorder of newborn related to fetal growth and fetal malnutrition | P05 |
|  | Extremely low birth weight (<1,000 g) | P070 |
|  | Low birth weight (1,000-2,499 g) | P071 |
|  | Extreme immaturity of newborn (< 28 weeks) | P072 |
|  | Preterm newborn (28-37 weeks) | P073 |
| Perinatal respiratory and cardiovascular disorder | Respiratory and cardiovascular disorder specific to the perinatal period | P20-P29 |
| Perinatal congenital malformation | Congenital malformation of the heart | Q20-Q24 |
|  | Congenital malformation of great vessels | Q25-Q26 |
| Perinatal congenital defect | Congenital defect originating in perinatal period | Q02, Q30, Q32-Q37, Q44, Q60, Q61, P70, P78 |
| Cardiovascular disorders | Cardiovascular disease | A520, I00-I02, I05-I11, I13, I15, I120-I28, I30-I52 P290, Q230-Q233, R000, R001, R008, T821, Z450, Z95 |
| Haematological disorders | Blood disease | D50, D51-D53, D65-D69 |
|  | Sickle-cell disorder | D57 |
| Hepatic disorders | Liver disease | B18, I85, I86, I98, K70-K72, K74, K76 |
| Infectious diseases | Tuberculosis | A15-A19 |
|  | HIV/AIDS | B20-B22, B24 |
| Solid organ transplant | Transplant | Z94 |
| Malignancies | Cancer | C00-C26, C30-C34, C37-C41, C43, C45-C58, C60-C85, C88, C90, C96, C97 |
| Metabolic disorders | Diabetes | E10-E14 |
| Musculoskeletal disorders | Polyarteritis nodosa | M30 |
| Neurodevelopment disorders | Down syndrome | Q90 |
| Neurological disorders | Neurological disease | G10-G13, G20, G22, G25, G31, G32, G35- G37, G40, G41, G93, R47, R56 |
| Nutritional disorders | Vitamin D deficiency | E55 |
| Pregnancy | Pregnancy | O00-O99 |
| Renal disorders | Renal failure | I12, I13, N18, N19, N25, Z49, Z99 |
| Respiratory disorders | Asthma | J45, J46 |
|  | Chronic obstructive pulmonary disease | J44 |
|  | Chronic pulmonary disease | I27, J40-J43, J47, J60-J68, J70 |
|  | Cystic fibrosis | E84 |

# Supplemental Table 2. High-risk categories for patients with respiratory syncytial virus infection by age group

| **High risk category** | **Examples** | | |
| --- | --- | --- | --- |
|  | **< 2 years** | **2-18 years** | **Adults (> 18)** |
| Prematurity | Small for gestational age, low birth weight, extremely low birth weight |  |  |
| Perinatal respiratory and cardiovascular disorder | Bronchopulmonary dysplasia, transient tachypnea of newborn | Bronchopulmonary dysplasia |  |
| Perinatal congenital malformation | Cardiac septal defect, atent ductos arteriosus, | Cardiac septal defect, atent ductos arteriosus, |  |
| Perinatal congenital defect | Atresia of bile ducts, cleft palate | Atresia of bile ducts, cleft palate |  |
| Cardiovascular disorders | Cardiac septal defect, CHF | Hypertension, CHF | Hypertension, AF |
| Haematological disorders | Thrombocytopenia, iron deficiency anemia | Thrombocytopenia, iron deficiency anemia | Thrombocytopenia, iron deficiency anemia |
| Hepatic disorders | Biliary cirrhosis, portal hypertension | Biliary cirrhosis, portal hypertension | HBV, alcoholic cirrhosis |
| Infectious diseases | HIV, tuberculosis | HIV, tuberculosis | HIV, tuberculosis |
| Solid organ transplant |  | Kidney transplant | Kidney transplant |
| Malignancies | Lymphoblastic leukaemia | Lymphoblastic leukaemia | Lymphoblastic leukaemia |
| Metabolic disorders |  | DM | DM |
| Musculoskeletal disorders | Mucocutaneous lymph node syndrome | Mucocutaneous lymph node syndrome | Polyarteritis |
| Neurodevelopment disorders | Down’s syndrome | Down’s syndrome | Down’s syndrome |
| Neurological disorders | Epilepsy, febrile convulsion | Epilepsy, multiple sclerosis | Parkinson’s disease, epilepsy |
| Nutritional disorders | Vitamin D deficiency | Vitamin D deficiency | Vitamin D deficiency |
| Pregnancy |  |  | Pregnancy |
| Renal disorders | Renal failure | CKD | CKD, hypertensive renal disease |
| Respiratory disorders | Asthma, bronchitis | Asthma, bronchitis | Asthma, COPD |
